# Optimizing the Boosting Schedule of Subunit Vaccines Consisting of BCG and “Non-BCG” Antigens to Induce Long-Term Immune Memory

**DOI:** 10.3389/fimmu.2022.862726

**Published:** 2022-04-12

**Authors:** Wei Lv, Pu He, Yanlin Ma, Daquan Tan, Fei Li, Tao Xie, Jiangyuan Han, Juan Wang, Youjun Mi, Hongxia Niu, Bingdong Zhu

**Affiliations:** ^1^ Gansu Provincial Key Laboratory of Evidence Based Medicine and Clinical Translation and Lanzhou Center for Tuberculosis Research, Institute of Pathogen Biology, School of Basic Medical Sciences, Lanzhou University, Lanzhou, China; ^2^ Institute of Pathophysiology, School of Basic Medical Sciences, Lanzhou University, Lanzhou, China; ^3^ State Key Laboratory of Veterinary Etiological Biology, College of Veterinary Medicine, Lanzhou University, Lanzhou, China

**Keywords:** tuberculosis, BCG, subunit vaccine, boost schedule, immunization program

## Abstract

Boosting Bacillus Calmette-Guérin (BCG) with subunit vaccine is expected to induce long-term protection against tuberculosis (TB). However, it is urgently needed to optimize the boosting schedule of subunit vaccines, which consists of antigens from or not from BCG, to induce long-term immune memory. To address it two subunit vaccines, Mtb10.4-HspX (MH) consisting of BCG antigens and ESAT6-CFP10 (EC) consisting of antigens from the region of difference (RD) of *Mycobacterium tuberculosis* (*M. tuberculosis*), were applied to immunize BCG-primed C57BL/6 mice twice or thrice with different intervals, respectively. The long-term antigen-specific immune responses and protective efficacy against *M. tuberculosis* H37Ra were determined. The results showed that following BCG priming, MH boosting twice at 12-24 weeks or EC immunizations thrice at 12-16-24 weeks enhanced the number and function of long-lived memory T cells with improved protection against H37Ra, while MH boosting thrice at 12-16-24 weeks or twice at 8-14 weeks and EC immunizations twice at 12-24 weeks or thrice at 8-10-14 weeks didn’t induce long-term immunity. It suggests that following BCG priming, both BCG antigens MH boosting twice and “non-BCG” antigens EC immunizations thrice at suitable intervals induce long-lived memory T cell-mediated immunity.

## 1 Introduction

Tuberculosis (TB) is a serious infectious disease mainly caused by *Mycobacterium tuberculosis* (*M. tuberculosis*) (1, 2). Bacillus Calmette-Guerin (BCG) is commonly applied in newborns and has proved to be effective in protecting children from severe tuberculosis infection (3, 4), but the protective immunity wanes and shows limited protection against tuberculosis in adults (5, 6). T cell-mediated immune responses are critical for host defense against *M. tuberculosis* infection (7–9). *M. tuberculosis* infection or vaccine immunization activates several kinds of T cells, including stem cell-like memory T cells (T_SCM_), central memory T cells (T_CM_), effector memory T cells (T_EM_) and effector T cells (T_eff_) (10, 11). T_SCM_ and T_CM_ can be maintained for many years and have strong proliferation ability, in which T_SCM_ can live longer than T_CM_ (8, 12–14). Under secondary infection or antigen re-stimulation, T_SCM_ could differentiate into T_CM_, which mainly secrete interleukin-2 (IL-2); T_CM_ can differentiate into T_EM_ and T_eff_, and then secrete cytokine interferon-γ (IFN-γ) (13, 15, 16). T_EM_ can be maintained for 4 to 8 weeks and provides short-term protection (17). Therefore, long-term protection against TB depends on long-lived memory T cells such as T_SCM_ and T_CM_ cells (18, 19).

BCG mainly induces T_EM_ which may wane as the children grow up, failing to provide enough protection in adults (20). It is supposed that adjuvanted subunit vaccine had the potential to boost BCG-primed immunity. For example, ten weeks after BCG priming, BALB/c mice received ChAdOx1.85A or MVA85A vaccine twice every 4 weeks showed significantly decreased bacterial burdens in the lung when the mice were challenged with aerosolized *M. tuberculosis* 4 weeks after the last immunization (*P* < 0.01) (21). In addition, H4-IC31H vaccine boosting at 19^th^ and 22^nd^ week after BCG immunizations significantly reduced bacterial burdens in lung and spleen compared with BCG when the mice were challenged with *M. tuberculosis* 5 weeks after the final booster vaccination (22). Cynomolgus macaques were primed with BCG and boosted with Mtb72F/AS02A three times with 4 weeks apart induced protection superior to BCG alone when the monkeys were challenged with *M. tuberculosis* 4 weeks after the last immunization (23).

However, the above traditional short-interval subunit vaccine boosting programs usually induced T_EM_ and provided short-term protection (24, 25). It was reported that macaques vaccinated with BCG and boosted with M72 vaccine at 16 and 20 weeks did not enhance the protective efficacy of BCG when the macaques were challenged with a low dose (8–16 CFU) of *M. tuberculosis* Erdman *via* bronchoscope at 12 weeks after the final immunization (26). In a clinical trial, boosting healthy infants (aged 4-6 months) who received BCG previously with MAV85A induced modest cell-mediated immune responses without improving protective efficacy against TB (27). Besides limited antigen profile, the boosting progress was supposed to be a reason for the poor protection (28–31). Therefore, how to boost BCG-primed immune memory with subunit vaccine to induce long-lived memory T cells is urgently needed to be investigated.

It is well-known that antigen stimulation times and intervals might affect the development of T_CM_ (32, 33). As far as subunit vaccine immunization schedule was considered, our previous work found that compared with the traditional immunization program of 0-3-6 weeks, prolonging the intervals of immunization, the schedule of 0-4-12 weeks, could increase the number and function of long-lived memory T cells and improve the protective efficacy (25).

The immunization program of boosting BCG with subunit vaccine is complicated. We hypothesized that BCG antigens and “non-BCG” antigens (being absent from BCG), from RD of *M. tuberculosis*, might require different boosting programs to induce long-term immune memory. In this experiment, Mtb10.4-HspX (MH) protein was used as the representative of antigen from BCG and ESAT6-CFP10 (EC) protein was applied as the representative antigen from RD (34, 35). The optimized immunization schedules for these two types of antigens were investigated.

## 2 Materials and Methods

### 2.1 Animals and Ethics Statement

Specific pathogen-free 6-8-week-old female C57BL/6J mice were purchased from Gansu University of Chinese Medicine (Lanzhou, China). Animals received free access to water and standard mouse chow throughout the study. All animal experiments were carried out under the guidelines of the Council on Animal Care and Use, and the protocols were reviewed and approved by the Institutional Animal Care and Use Committee of Lanzhou University.

### 2.2 Preparation of H37Ra, BCG and Antigen Proteins

*M. tuberculosis* H37Ra (ATCC25177) and BCG (Danish strain) bacteria were cultured in Sauton’s medium. The fusion proteins MH and EC were prepared as previously reported (35, 36). In brief, MH fusion antigen without tag was purified by hydrophobic interaction chromatography using butyl-sepherose high performance (Butyl HP) column and ion-exchange chromatography using Q-sepharose high performance (Q HP) column (35). The fusion antigen EC without tag was purified by ion-exchange chromatography using Q HP column (36). Single mycobacterial proteins heat shock protein X (HspX), 10 kDa culture filtrate protein (CFP10) and 6 kDa early secreted antigen target (ESAT6) with His tag were purified by Ni-NTA His column (Novagen) (37, 38). The endotoxin concentrations of fusion protein were tested by Limulus amebocyte lysate (LAL) (Xiamen bioendo technology co., ltd, Xiamen, China). The purified protein derivative (PPD) of tuberculin was extracted from BCG, which contained a variety of proteins with different molecular weights.

### 2.3 Vaccine Immunization Program

#### 2.3.1 Long Interval Immunization Schedule

**Figure 1:**
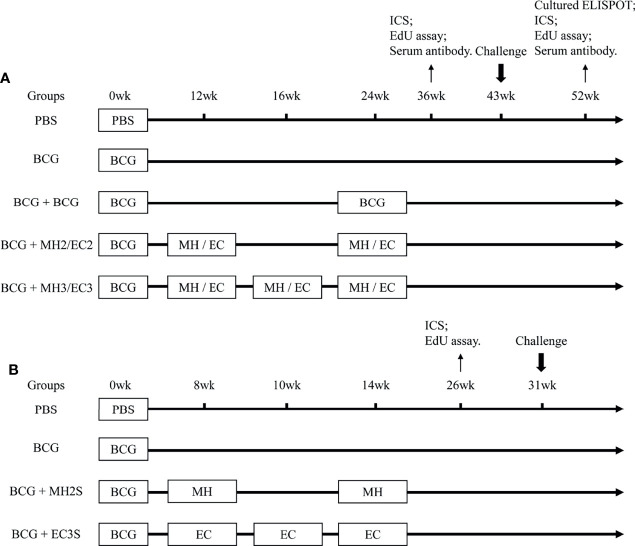
Immunization Schedule. **(A)** Long interval immunization schedule: C57BL/6 mice were primed with BCG and boosted with MH/EC at 12-24 weeks and 12-16-24 weeks respectively. Then, the function of memory T cells was evaluated at 12 weeks and 28 weeks after the last immunization. At 19 weeks after the last immunization, the mice were challenged intranasally with H37Ra, and lung tissues were collected for CFU counting 3 weeks after the challenge. **(B)** Short interval immunization schedule: C57BL/6 mice were primed with BCG and immunized with MH at 8-14 weeks or EC at 8-10-14 weeks respectively. The immune response of memory T cells was evaluated at 12 weeks after the last immunization. At 17 weeks after the last immunization, mice were challenged intranasally with H37Ra, and lung tissues were collected for CFU counting 3 weeks after the challenge. ICS, intracellular cytokine staining.

The mice were primed subcutaneously with BCG (5 × 10^5^ CFU in 100 μl per mouse). The purified protein MH or EC (10μg/dose) was emulsified in an adjuvant being composed of N, N′-dimethyl-N, N′-dioctadecyl ammonium bromide (DDA) (250μg/dose) (Anhui Super chemical technology Co., Ltd., China) and polyinosinic-polycytidylic acid (Poly I: C) (50μg/dose) (Kaiping Genuine Biochemical Pharmaceutical Co., Ltd., Guangdong, China) to construct subunit vaccine (39). To observe the long-term immune memory and protective efficacy, the BCG-primed mice were boosted with MH and EC twice or thrice subcutaneously with a long interval: MH/EC immunizations at 12-24 weeks groups; MH/EC immunizations at 12-16-24 weeks groups. The number of mice per group was 30. The BCG-primed mice were revaccinated subcutaneously with BCG (5 × 10^5^ CFU in 100 μl per mouse) at 24 weeks to be consistent with other boosters on immunoassays. BCG is a live attenuated tuberculosis vaccine and is usually boosted once when considering re-vaccination (40). PBS and BCG without boosting groups were used as control (**Figure 1A**). The immune memory was evaluated at 12 weeks and 28 weeks after the last immunization. The long-term protective efficacy was detected by H37Ra (5 × 10^6^ CFU in 50 μl per mouse) intranasal challenge at 19 weeks after the last immunization.

#### 2.3.2 Short Interval Immunization Schedule

Based on the results from the long interval immunization schedule, the protective efficacy of subunit vaccine boosting with a shortened interval was observed. The BCG-immunized mice were boosted with MH at 8-14 weeks or EC at 8-10-14 weeks subcutaneously (*SC*). The number of mice per group was 10. PBS and BCG without boosting groups were used as control (**Figure 1B**). The immune responses were detected by flow cytometry at 12 weeks after the last immunization. The long-term protective efficacy was detected by H37Ra intranasal challenge at 17 weeks after the last immunization.

### 2.4 Flow Cytometry and Intracellular Cytokine Staining (ICS)

#### 2.4.1 IFN-γ and IL-2 Secretion Following Antigen Stimulation *In Vitro*

Lymphocytes were isolated from spleen or bone marrow of mice by using Mouse 1 × Lymphocyte Separation Medium (Dakewe Biotech Company Limited, China) and cultured in media containing RPMI-1640, 10% fetal bovine serum (FBS), 100 U/ml Penicillin-Streptomycin Solution. Then the lymphocytes were inoculated in 24 well plates at the number of 5 × 10^6^ cells/well. The lymphocytes were stimulated with PPD (4 ug/ml) or mixed antigens PHEC including PPD (4 ug/ml), HspX (2ug/ml), ESAT6 (2ug/ml) and CFP10 (2ug/ml) at 37°C, 5% CO_2_. To keep experiments consistent, the same stimulus (PHEC) was used to observe the immune responses induced by the different vaccines. After 4 hours of stimulation, the cells were incubated for 7-8h with BD GolgiPlug™ (containing brefeldin A) at 37°C, 5% CO_2_. At last, the cells were collected and stained with anti-CD4-FITC (RM4-5, eBioscience) and anti-CD8-PerCP-Cy5.5 (53-6.7, eBioscience). Lymphocytes were permeabilized using the BD Cytofix/Cytoperm kit according to the manufacturer’s instructions and stained with anti-IFN-γ-APC (XMG1.2, eBioscience) and anti-IL-2-PE (JES6-5H4, BD). Lymphocytes from individual mice were analyzed on a NovoCyte flow cytometer (ACEA Biosciences). Flow cytometry gating strategy was shown in **Supplementary Figure 1A**. The spleen or bone marrow lymphocytes were first gated by the parameters SSC-H and FSC-H (lymphocytes), and then single cells were gated by the parameters FSC-H and FSC-A (single cells). Finally, CD4^+^ IFN-γ^+^ T cells, CD4^+^ IL-2^+^ T cells, CD8^+^ IFN-γ^+^ T cells, CD8^+^ IL-2^+^ T cells or CD8^+^ IFN-γ^+^ IL-2^+^ T cells were analyzed by flow cytometric. The analyzed cytokine-producing T-cells were either represented as percentages among total number of spleen lymphocytes or described as actual counts of cytokine-producing cells as bar graphs.

#### 2.4.2 IFN-γ Secretion Following Re-Stimulation With Antigens *In Vivo* and *In Vitro*

Our previous experiment showed that at 25 weeks following subunit vaccine immunization, once antigen stimulation could not induce cytokines production (41), which suggests that T_EM_ cells wane at that time (42). Therefore, at 28 weeks after vaccination only long-lived memory T cells (T_SCM_ and T_CM_) exist. Since the percentage of antigen-specific long-lived memory T cells was too few to be detected directly by the surface markers of central memory T cells (CCR7, CD62L, CD44, and CD127) (17). Upon antigen stimulation antigen-specific T_SCM_ and T_CM_ cells proliferate and differentiate into T_EM_ and T_eff_ cells, and then produce cytokine IFN-γ (32, 43, 44). Based on the principle, in our previous studies we detected the role long-lived memory T cells through analyzing IFN-γ production following stimulation with same antigen twice every 9 days (25, 41).

At 28 weeks after last immunization, the vaccine-immunized mice were injected subcutaneously with mixed of antigens of PPD (4 ug/mouse), HspX, EAST6, CFP10 (2 ug/mouse of each protein) *in vivo*. The long-lived memory T cells were supposed to be activated and differentiated into T_EM_ or T_eff_ cells (32). Subsequently, the spleen lymphocytes were separated at 3 days later and stimulated with the same mixed antigens of PPD (4 ug/ml), HspX, EAST6, CFP10 (2 ug/ml of each protein) for 12 hours *in vitro*, during that time T_EM_ cells could differentiate into T_eff_ cells and produce IFN-γ. The intracellular cytokine staining was analyzed by flow cytometry to indirectly reflect the function of long-lived memory T cells (25, 45).

### 2.5 Cultured IFN-γ ELISPOT Assay

A cultured IFN-γ enzyme-linked immunospot (ELISPOT) assay was also used to evaluate the immune responses of long-lived memory T cells (43, 44). Twenty-eight weeks after the last immunization, spleen lymphocytes were suspended in RPMI-1640 medium supplemented with 10% fetal bovine serum, 100U/ml Penicillin-Streptomycin Solution, 2 mM L-glutamine, 25 mM HEPES buffer, 1% sodium pyruvate, and 50 mM 2-mercaptoethanol. Spleen lymphocytes (5 × 10^6^ cells/ml/well) were stimulated with mixed antigens of PHEC containing PPD (4 μg/ml) and HspX, ESAT-6, CFP10 (2 μg/ml of each protein). Spleen lymphocytes were incubated at 37°C and 5% CO_2_ with half culture media containing recombinant human IL-2 (rhIL-2) 100 U/ml, which were replaced on days 3 and 7, allowing expansion of antigen-specific T cells. On day 9, the cultured cells were harvested and antigen-presenting cells (APCs) were added. Then, cultured cells were plated (1 × 10^6^ cells/well) and re-stimulated with PHEC for an additional 20 hours in the presence of APCs in anti-IFN-γ coated ELISPOT plates (Dakewe Biotech Company Limited, China). The spot-forming cells (SFCs) were counted by an ELISPOT reader (Dakewe Biotech Company Limited, China).

### 2.6 EdU Proliferation Assay for Long-Lived Memory T Cells

5-Ethynyl-2’-deoxyuridine (EdU) is to be infiltrated into the deoxyribonucleic acid (DNA) of T cells as the cells proliferate, and it can be detected following proliferation and division of memory T cells. Spleen lymphocytes (5 × 10^6^ cells/well) were stimulated with the mixed antigens PHEC for 7 days in 24-well plates. Three days after antigen stimulation, when T_SCM_ and T_CM_ were to be activated into T_EM_, EdU (Click-iT™ EdU Flow Cytometry Assay Kit, Invitrogen™, OR, USA) was added at a final concentration of 30 μM and the lymphocytes were continued to be cultured for 4 days. On day 7, cells were harvested, fixed, permeabilized, and incubated with Click-iT reaction buffer according to the manufacturer’s instructions of the Click-iT™ EdU Flow Cytometry Assay Kit. Subsequently, cells were stained with anti-CD4-APC (RM4-5, eBioscience). Finally, a flow cytometry assay was performed to evaluate the proliferating capability of CD4 ^+^ T cells. Spleen lymphocytes were first gated by the parameters SSC-H and FSC-H (lymphocytes), and then single cells were gated by the parameters FSC-H and FSC-A (single cells). Flow cytometry gating strategy was shown in **Supplementary Figure 1B**.

### 2.7 Detection of Antigen-Specific Antibodies in Mouse Sera by ELISA

At 12 and 28 weeks after the last immunization, antigen-specific immunoglobulin IgG, IgG1, and IgG2c in sera were detected by enzyme-linked immunosorbent assay (ELISA). Firstly, 0.5 µg/well of PPD, HspX, and ESAT6 were separately added into the plate at 4°C overnight. Secondly, the plates were blocked with 5% skimmed milk powder, then incubated with the double-diluted serum at 37°C for an hour. And then the plates were washed and added 100 µL of goat anti-mouse IgG (Solarbio, Beijing, China) and rabbit anti-mouse IgG1 and IgG2c (Rockland Immunochemicals Inc., Montgomery, PA, USA) The 3,3′,5,5′-tetramethylbenzidine (TMB) substrate was added at 200 µL/well and incubated at room temperature for 5 min. The reaction was then stopped by diluted sulfuric acid (1 mol/L) at 50 µL/well. The color was quantified at 450 nm. The serum in the PBS group was used as the negative control. The antibody titer was evaluated as a reciprocal of each endpoint dilution.

### 2.8 Quantification of CFU of *Mycobacterium tuberculosis* H37Ra in Lung Tissue

The mice received intraperitoneal anesthesia with 1% sodium pentobarbital at a concentration of 50 mg/kg. Mice from each group were challenged through intranasal route (*i.n.*) with 5 × 10^6^ CFU of H37Ra. Lungs of infected animals were harvested three weeks after the H37Ra intranasal challenge. Organs were ground and resuspended in PBS. The dilutions were plated in Middlebrook 7H10 plates (BD) containing oleic acid/albumin/dextrose/catalase (OADC). The colony-forming units (CFU) were counted.

### 2.9 Statistical Analysis

The experimental data were expressed as Mean ± SD. The data were evaluated by GraphPad Prism 8.0 software with unpaired two-tailed Student′s *t*-tests to compare two groups and one-way analysis of variance (ANOVA) followed by a Tukey *post hoc* test to compare multiple groups. Among them, *P* < 0.05 was considered statistically significant.

## 3 Results

### 3.1 Longitudinal Changes of Immune Responses Induced by BCG Vaccination

To observe the longitudinal changes of immune responses induced by BCG, lymphocytes in spleen and bone marrow were stimulated with PPD antigen for 12 hours *in vitro* at different times after BCG immunization, and flow cytometry was used to quantify IFN-γ producing CD4^+^ T cells. In spleen lymphocytes, the frequency of IFN-γ producing CD4^+^ T cells peaked at 4 weeks, slightly decreased at 9 weeks, and the immune responses reduced to a low level at 12 weeks (**Supplementary Figures 2A, C**). In bone marrow, the frequency of IFN-γ producing CD4^+^ T cells increased at 4 weeks, reached the highest level at 9 weeks and decreased at 12 weeks (**Supplementary Figures 2B, D**). It indicated that the immune response induced by effector memory T cells decreased at the 12th week after BCG vaccination, which should be an optimal time for boosting (46–49).

### 3.2 Boosting BCG With Subunit Vaccines With Suitable Schedules Induced Long-Lived Memory T Cells

#### 3.2.1 Cytokines Production by Memory T Cells at 12 Weeks After Last Vaccine Immunization

The fusion proteins MH and EC and single proteins ESAT6, CFP-10 and HspX were prepared (**Supplementary Figure 3**). At the concentration of MH and EC (1mg/ml of each protein), the endotoxin levels were 0.009031EU/μg and 0.00985 EU/μg, respectively (**Supplementary Table 1**). According to above results, the mice were boosted with the subunit vaccine at 12 weeks after BCG immunization. Fusion protein EC consisting of RD antigen (“non-BCG” antigen) and MH consisting of BCG antigen were administered twice at 12-24 weeks and thrice at 12-16-24 weeks respectively to investigate which immunization programs could induce long-term immune memory to prolong BCG-primed immune responses (**Figure 1A**).

**Figure 2:**
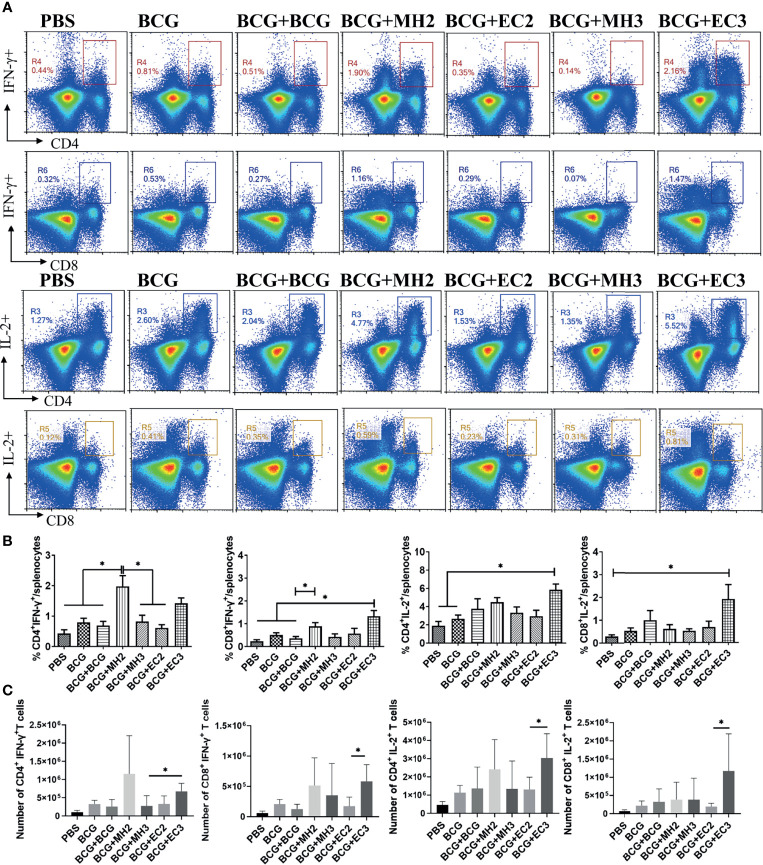
The ratio and number of IFN-γ and IL-2 producing T cells following antigen stimulation. At 12 weeks after the last immunization, the splenic lymphocytes were separated and stimulated with mixed antigens of PPD, ESAT-6, CFP10 and HspX (PHEC) *in vitro* for 12 hours. The intracellular cytokines staining was analyzed using flow cytometry. **(A)** Flow cytometric analysis of IFN-γ and IL-2 producing CD4^+^ T cells and CD8^+^ T cells. **(B)** Statistical analysis of the proportion of IFN-γ and IL-2 producing CD4^+^ T cells and CD8^+^ T cells. **(C)** Statistical analysis of the number of IFN-γ and IL-2 producing CD4^+^ T cells and CD8^+^ T cells among total spleen lymphocytes from each immunized group. Results are presented as means ± SD, *n* = 4 ~ 5. The data were evaluated with unpaired two-tailed Student′s *t*-tests to compare two groups and one-way analysis of variance (ANOVA) followed by a Tukey *post hoc* test to compare multiple groups. *P < 0.05.

To assess the frequency of vaccine-induced antigen-specific memory T cells at 12 weeks after last immunization, cytokines production in the splenocytes following specific antigens PHEC stimulation for 12 hours *in vitro* was analyzed by flow cytometry. The results showed that compared with MH boosting at 12-16-24 weeks group (0.82 ± 0.46) and EC immunizations at 12-24 weeks group (0.61 ± 0.21), MH boosting at 12-24 weeks group produced a higher frequency of IFN-γ producing CD4^+^ T cells (1.97 ± 0.70, *P* < 0.05; **Figures 2A, B**). Compared with PBS group (0.23 ± 0.12) and BCG revaccination group (0.36 ± 0.15), MH boosting at 12-24 weeks group (0.88 ± 0.32) and EC immunizations at 12-16-24 weeks group (1.33 ± 0.56) produced a higher frequency of IFN-γ producing CD8^+^ T cells (*P* < 0.05; **Figures 2A, B**). Compared with PBS (1.89 ± 0.95) group and BCG group (2.67 ± 0.7), EC immunizations at 12-16-24 weeks group (5.84 ± 1.41) increased frequency of IL-2 producing CD4^+^ T cells (**Figures 2A, B**). Furthermore, compared with MH boosting at 12-16-24 weeks group, the EC immunizations at 12-16-24 weeks group had a higher number of IFN-γ/IL-2 producing CD4^+^ and CD8^+^ T cells (*P* < 0.05; **Figure 2C**). Moreover, compared with MH boosting twice or EC immunizations thrice groups, MH boosting at 12-24 weeks and EC immunizations at 12-16-24 weeks improved the proportion of IFN-γ^+^ and IL-2^+^ double-positive CD8^+^ T cells (**Supplementary Figure 4**). The results indicated that MH boosting at 12-24 weeks and EC immunizations at 12-16-24 weeks induced strong memory T cell-mediated immune response compared with the EC immunizations at 12-24 weeks and MH boosting at 12-16-24 weeks.

#### 3.2.2 Antigen-Specific Cytokines Production by Long-Lived Memory T Cells at 28 Weeks After the Last Vaccine Immunization

At 28 weeks after vaccine immunization, the antigen-specific effector memory T cells would fade away, so the vaccine-induced long-lived memory T cells were analyzed (41). The immune responses following antigen stimulation to monitor the number and function of vaccine-induced long-lived memory T cells indirectly by two methods as follow (25, 41).

**Figure 3:**
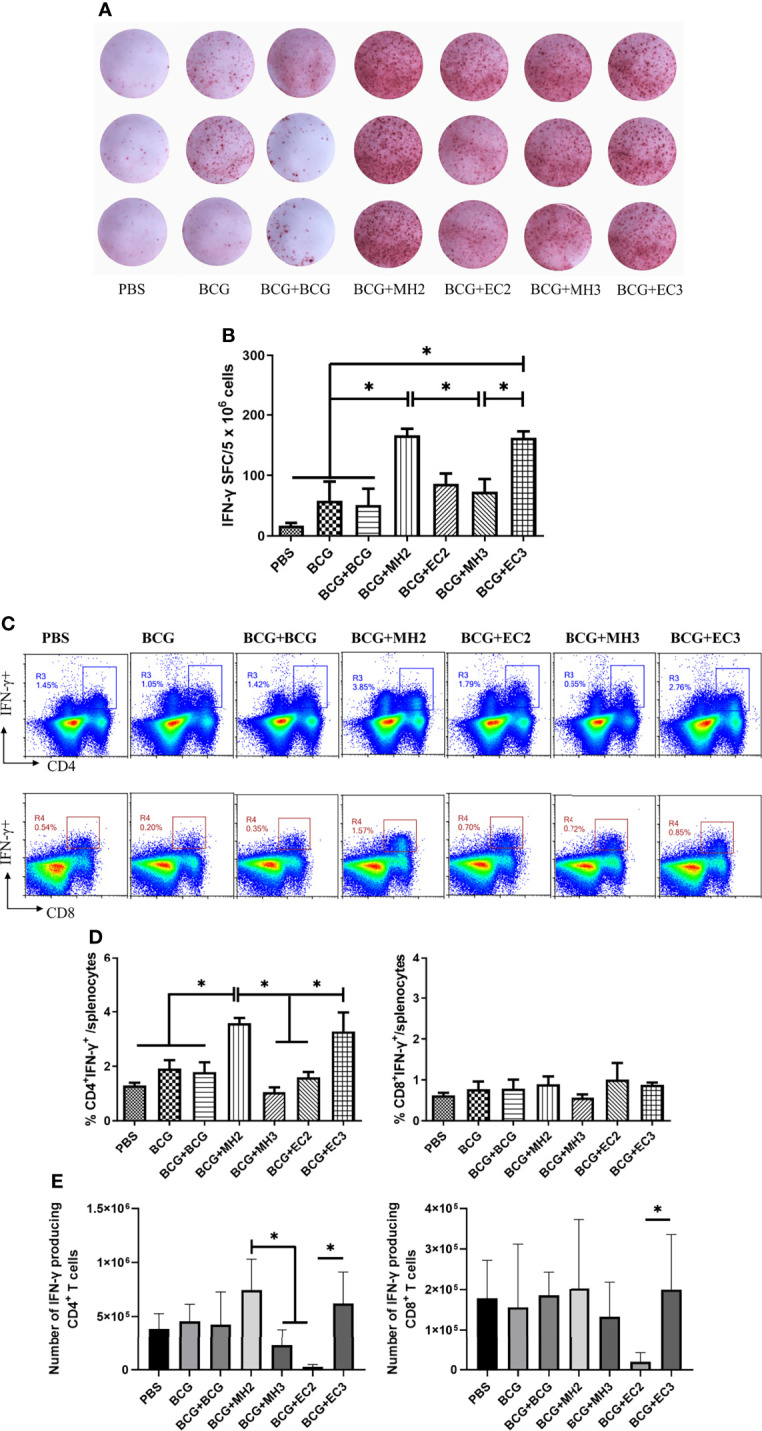
IFN-γ production by long-lived memory T cells. At 28 weeks after the last immunization, spleen lymphocytes were cultured with or without mixed antigens of PPD, HspX, ESAT-6, and CFP10 for 9 days and then the cells were harvested and restimulated with the same antigens for 20 hours in the presence of APCs in anti-IFN-γ coated ELISPOT plates. At 28 weeks after the last immunization, mice were injected subcutaneously with mixed antigens of PPD, ESAT-6, CFP10 and HspX (PHEC) for 3 days. Then, mice were euthanized and spleen lymphocytes were isolated and stimulated with mixed antigens PHEC for 12 hours *in vitro* and analyzed by Flow cytometry. **(A)** Representative images of IFN-γ ELISPOT wells from long-term cultured IFN-γ ELISPOT assays. **(B)** Statistical analysis of the cultured IFN-γ ELISPOT assay. Results are presented as means ± SD, *n* = 4. *P < 0.05. **(C)** Flow cytometric analysis of IFN-γ producing CD4^+^ and CD8^+^ T cells. **(D)** Statistical analysis of the proportion of IFN-γ producing CD4^+^ T cells and CD8^+^ T cells. **(E)** Statistical analysis of the actual number of IFN-γ producing T cells among total spleen lymphocytes from each immunized group. Results are presented as means ± SD, *n* = 4 ~ 5. The data were evaluated with unpaired two-tailed Student′s *t*-tests to compare two groups and one-way analysis of variance (ANOVA) followed by a Tukey *post hoc* test to compare multiple groups. *P < 0.05.

First, the cultured ELISPOT was used to investigate the function and number of antigen-specific long-lived memory T cells (43). The results showed that compared with the group of EC immunizations at 12-24 weeks (86.2 ± 33.7 SFCs/5 × 10^6^ cells), MH boosting at 12-24 weeks group induced an increasing number of antigen-specific IFN-γ producing T cells (166 ± 22.4 SFCs/5 × 10^6^ cells, *P <* 0.05; **Figures 5A, B**). In addition, compared with the group of MH boosting at 12-16-24 weeks (73 ± 52 SFCs/5 × 10^6^ cells), the group of EC immunizations at 12-16-24 weeks (162.25 ± 18.3 SFCs/5 × 10^6^ cells) significantly elevated numbers of antigen-specific IFN-γ secreting T cells (*P <* 0.05; **Figures 3A, B**). The results indicated that both MH boosting at 12-24 weeks and EC immunizations at 12-16-24 weeks might induce a great number of long-lived memory T cells.

Second, according to the principle of cultured ELISPOT, flow cytometry assay was used to detect the immune responses of long-lived memory T cells under repeated antigen stimulation (25, 43). The immunized mice were stimulated subcutaneously with PHEC antigens *in vivo* 3 days prior to immunoassay. It was supposed that the long-lived memory T cells activated and developed into T_EM_ or T_eff_. Three days later, spleen lymphocytes were isolated and stimulated with PHEC antigens for 12 hours *in vitro*, during that time the T_EM_ developed into T_eff_ and secreted cytokine IFN-γ. Then the secretion of cytokines IFN-γ was detected by intracellular cytokine staining. This method indirectly reflected the functions of vaccine-induced long-lived memory T cells (11, 25). The results showed that compared with PBS group (1.29 ± 0.17), BCG group (1.91 ± 0.53), and the groups of EC immunizations twice at 12-24 weeks (1.59 ± 0.34) and MH boosting thrice at 12-16-24 weeks (1.00 ± 0.38), MH boosting twice at 12-24 weeks group (3.58 ± 0.37) produced high frequencies of IFN-γ producing CD4^+^ T cells (*P* < 0.05; **Figures 3C, D**). Compared with the group of MH boosting thrice at 12-16-24 weeks (1.00 ± 0.38), EC immunizations thrice at 12-16-24 weeks group produced higher frequencies of IFN-γ producing CD4^+^ T cells (3.27 ± 1.21, *P* < 0.05; **Figures 3C, D**). The MH boosting at 12-24 weeks group had a higher number of IFN-γ producing CD4^+^ T cells compared with EC immunizations at 12-24 weeks group (*P* < 0.05; **Figure 3E**). The EC immunizations at 12-16-24 weeks group had more IFN-γ producing CD4^+^ T cells compared with EC immunizations at 12-24 weeks group (*P* < 0.05; **Figure 3E**). The above results indicated that MH boosting twice at 12-24 weeks and EC immunizations thrice at 12-16-24 weeks increased the immune responses of long-lived memory T cells.

#### 3.2.3 The Proliferation Capability of Long-Lived Memory T Cells

**Figure 4:**
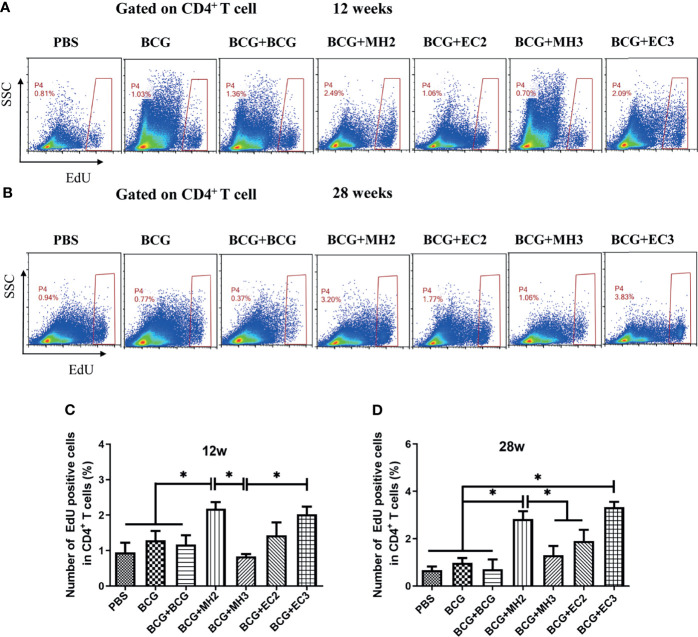
CD4 ^+^ T cell proliferation detected by EdU assay. At 12 weeks and 28 weeks after the last immunization, splenic lymphocytes (5×10^6^ cells/well) were stimulated with mixture antigens of PPD, ESAT-6, CFP10 and HspX *in vitro* for 7 days. Three days after antigen stimulation, EdU was added at a final concentration of 30 μM, continued to culture for 4 days and was determined using flow cytometry. **(A, B)** Representative experiments of flow cytometric analysis of CD4^+^ T cells proliferation. **(C)** Statistical analysis of CD4^+^ T cell proliferation at 12 weeks after the last immunization. **(D)** Statistical analysis of CD4^+^ T cell proliferation at 28 weeks after the last immunization. Results are presented as means ± SD, *n* = 4 ~ 5. The data were evaluated with unpaired two-tailed Student′s *t*-tests to compare two groups and one-way analysis of variance (ANOVA) followed by a Tukey *post hoc* test to compare multiple groups. *P < 0.05.

To verify the proliferative capacity of T cells induced by different boosting programs, the proliferation capacity of long-lived memory T cells was analyzed by the EdU method at 12 weeks and 28 weeks after the last immunization, respectively. At 12 weeks after the last immunization, compared with PBS group (0.95 ± 0.46), BCG group (1.28 ± 0.46), and BCG revaccination group (1.17 ± 0.45), the proportion of EdU^+^ cells in the MH boosting twice at 12-24 weeks group (2.18 ± 0.32) increased significantly *(P* < 0.05). The proportion of EdU^+^ cells in MH boosting twice at 12-24 weeks group (2.18 ± 0.32) and EC immunizations thrice at 12-16-24 weeks group (2.00 ± 0.36) were significantly higher than MH boosting thrice at 12-16-24 weeks group (1.42 ± 0.63) (*P* < 0.05; **Figures 4A, C**). At 28 weeks after the last immunization, compared with the PBS group (0.67 ± 0.31), BCG group (0.98 ± 0.40) and BCG revaccination group (0.71 ± 0.57), the proportion of EdU^+^ cells in MH boosting twice at 12-24 weeks group (2.83 ± 0.57) and EC immunizations thrice at 12-16-24 weeks group (3.33 ± 0.39) increased significantly *(P* < 0.05; **Figures 4B, D**). Compared with the MH boosting thrice at 12-16-24 weeks group (1.20 ± 0.78), and EC immunizations twice at 12-24 weeks group (1.89 ± 0.82), the proportion of EdU^+^ cells in MH boosting twice at 12-24 weeks group (2.83 ± 0.57) increased significantly *(P* < 0.05; **Figures 4B, D**). Taken together, both MH boosting twice at 12-24 weeks group and EC immunizations thrice at 12-16-24 weeks group enhanced the proliferative capacity of CD4^+^ T cells.

### 3.3 BCG-Prime and MH/EC-Boost Induced Durable Humoral Immune Response

At 12 weeks and 28 weeks after the last immunization, the IgG, IgG1 and IgG2c against HspX, ESAT6 and PPD in serum were measured by ELISA. The results demonstrated that compared with BCG and BCG revaccination groups, MH/EC-boosting groups produced long-durable higher levels of antibody titers (*P* < 0.05; **Table 1**). Furthermore, MH/EC boosting at 12-16-24 weeks group produced significantly higher levels of HspX/ESAT6-specific IgG, IgG1, and IgG2c than MH/EC boosting at 12-24 weeks group (*P* < 0.05; **Table 1**). The results indicated that MH/EC vaccine boosting induced durable strong serum antibodies.

**Table 1:** The production of antigen-specific IgG, IgG1, and IgG2c.

	Groups	12weeks				28weeks			
		IgG	IgG1	IgG2c	IgG2c/IgG1	IgG	IgG1	IgG2c	IgG2c/IgG1
**Anti-HspX**	**BCG**	2.31 ± 0.19	2.33 ± 1.03	1.34 ± 0.41	0.57 ± 0.17	2.19 ± 0.12	0.74 ± 0.53	0.62 ± 0.3	0.92 ± 0.37
	**BCG+BCG**	2.28 ± 0.11	2.50 ± 0.17	1.27 ± 0.26	0.50 ± 0.1	1.84 ± 0.49	0.87 ± 0.59	0.80 ± 0.29	0.84 ± 0.45
	**BCG+MH2**	2.93 ± 0.27^*^	4.39 ± 0.67^*^	3.48 ± 0.31^*^	0.79 ± 0.07	2.91 ± 0.46^*^	3.12 ± 0.81^*^	2.83 ± 0.3^*^	0.90 ± 0.1
	**BCG+MH3**	4.00 ± 0.17^*^#^^	5.74 ± 0.7^*^#^^	4.84 ± 0.81^*^#^^	0.84 ± 0.14	3.78 ± 0.42^*^#^^	3.93 ± 0.44^*^#^^	3.6 ± 0.39^*^#^^	0.93 ± 0.1
**Anti-PPD**	**BCG**	3.00 ± 0.25	2.20 ± 0.13	2.67 ± 0.12	1.21 ± 0.05	2.28 ± 0.41	1.52 ± 0.1	1.66 ± 0.16	1.09 ± 0.1
	**BCG+BCG**	2.80 ± 0.51	2.30 ± 0.17	2.73 ± 0.1	1.18 ± 0.04	2.26 ± 0.32	1.47 ± 0.34	1.89 ± 0.35	1.28 ± 0.24
	**BCG+MH2**	3.20 ± 0.11^*^	3.71 ± 0.49^*^	3.96 ± 0.32^*^	1.10 ± 0.06	3.01 ± 0.4^*^	2.01 ± 0.32^*^	2.98 ± 0.37^*^	1.48 ± 0.18
	**BCG+MH3**	4.27 ± 0.37^*^#^^	5.40 ± 0.31^*^#^^	5.71 ± 0.54^*^#^^	1.07 ± 0.1	3.92 ± 0.9^*^#^^	5.13 ± 1.65^*^#^^	3.76 ± 0.44^*^#^^	0.73 ± 0.08
**Anti-ESAT6**	**BCG**	2.17 ± 0.32	1.96 ± 0.41	2.00 ± 0.31	0.84 ± 0.08	2.15 ± 0.14	1.00 ± 0.56	2.10 ± 0.37	2.18 ± 0.27
	**BCG+BCG**	2.19 ± 0.11	2.31 ± 0.18	1.94 ± 0.2	1.02 ± 0.16	2.05 ± 0.28	1.00 ± 0.37	1.98 ± 0.05	1.98 ± 0.05
	**BCG+EC2**	2.57 ± 0.19^*^	4.93 ± 0.78^*^	2.14 ± 0.75	0.43 ± 0.15	2.61 ± 0.06^*^	2.15 ± 0.63^*^	2.92 ± 0.53^*^	1.34 ± 0.28
	**BCG+EC3**	3.11 ± 0.35^*^$^^	5.63 ± 0.69^*^	2.57 ± 0.48	0.45 ± 0.08	2.83 ± 0.15^*^$^^	2.65 ± 0.51^*^	3.30 ± 0.11^*^	1.24 ± 0.04

At 12 weeks and 28 weeks after the last immunization, the IgG, IgG1 and IgG2c against HspX, PPD and ESAT6 in serum were measured by ELISA. Data are expressed as means ± standard deviation (SD) (n = 4). Antibody titers are presented as the means of log10 antibody titers ± SD. The statistical significance of data was determined using the unpaired two-tailed Student′s t‐test. *P < 0.05 vs. BCG plus BCG + BCG; ^#^P < 0.05 vs. BCG+MH2; and ^$^P < 0.05 vs. BCG+EC2.

### 3.4 The Protective Efficacy of BCG-Prime and MH/EC Boosting at Different Schedules

**Figure 5:**
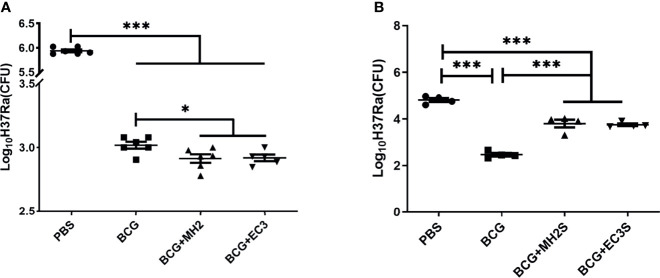
Bacterial burden at necropsy. **(A)** In the long interval immunization schedules, at 19 weeks after the last immunization, the immunized mice were challenged intranasally with *M. tuberculosis* H37Ra. **(B)** In the short interval immunization schedules, at 17 weeks after the last immunization, the immunized mice were challenged intranasally with *M. tuberculosis* H37Ra. At 3 weeks after challenge, mice were euthanized and the bacterial burden was measured in the lungs. Data were presented as log_10_ CFU ± SD from groups of 5-6 mice. The data were evaluated with unpaired two-tailed Student′s *t*-tests to compare two groups and one-way analysis of variance (ANOVA) followed by a Tukey *post hoc* test to compare multiple groups. *P < 0.05, ***P < 0.001.

Furthermore, we observed the long-term protective effect induced by MH boosting twice at 12-24 weeks and EC immunizations at 12-16-24 weeks. The attenuated *M. tuberculosis* H37Ra, which expresses all single proteins of Mtb10.4/HspX and ESAT-6/CFP-10 (50), was used to challenge the immunized mice. ESAT-6 expression in H37Ra was further confirmed by western blotting (**Supplementary Figure 5**). Considering H37Ra was an attenuated strain and could be cleared in mice around 4 weeks, the mice were challenged with high doses of H37Ra and determined the bacteria load in lung tissue at 3 weeks after the challenge. The immunized mice were challenged intranasally with avirulent *M. tuberculosis* H37Ra at 19 weeks after the last immunization. The results demonstrated that BCG group, MH and EC immunizations groups induced a significant reduction of mycobacterial loads in the lungs compared with PBS controls (*P* < 0.01). The groups of MH boosting at 12-24 weeks and EC immunizations at 12-16-24 weeks reduced bacteria load in lung tissues, declining approximately 0.1 log_10_ CFU compared with the BCG control group (*P* < 0.05) (**Figure 5A**). It showed that the schemes of MH boosting twice at 12-24 weeks and EC immunizations thrice at 12-16-24 weeks promoted long-lived memory T cells and improved protective efficacy.

Based on the results from the long interval immunizing schedule, shorten interval schedules were designed, and the protective efficacy and immune responses were analyzed. Following BCG priming, mice were immunized with MH/DP twice at 8-14 weeks and the EC/DP thrice at 8-10-14 weeks (**Figure 1B**). At 12 weeks after the last vaccine immunization, the immune response and T cell proliferation were detected by flow cytometry. At 17 weeks after the last vaccine immunization, the protective efficacy was detected by H37Ra challenge. The results showed that MH/DP boosting twice at 8-14 weeks and the EC/DP immunizing thrice at 8-10-14 weeks did not improve T cell proliferative capacity and BCG-primed immune protection, although an increased IFN-γ production following antigen stimulation was observed (**Figure 5B**; **Supplementary Figure 6**). The results suggest that subunit vaccines need suitable boosting schedules to induce long-term immune memory.

## 4 Discussion

In this study, we investigated the long-term immune memory induced by BCG priming and MH/EC vaccine boosting with different regimens. We found that MH boosting at 12-24 weeks and EC immunizations at 12-16-24 weeks enhanced the long-lived memory T cell-mediated immunity and improved protection efficiency of BCG, while MH boosting at 8-14 weeks and EC immunizations at 8-10-14 weeks reduced the long-term protective efficacy compared with BCG without boosting.

BCG vaccination mainly activates effector memory T cells, which cannot be maintained for a long time. In this experiment, T cell immune responses following BCG immunization declined at 9-12 weeks, which were consistent with the results in our laboratory′s previous work (25, 51). Besides, kinetics of BCG induced immune responses in the spleen of BALB/c mice at weeks 3, 6, and 10 found that T cell activation peaked at week 3 and gradually declined thereafter (52). In the C57BL/6 mouse model, the immune response of T lymphocytes collected for retro-orbital blood was peaked at 3 weeks and weakened at 5 weeks following BCG vaccination (53). Furthermore, in BCG-vaccinated mice, anti-mycobacterial T cell responses persisted for long period, peaked at 12-32 weeks, and waned gradually thereafter (54, 55). These studies suggest that the mouse strain, BCG vaccine strain, and antigen(s) used for *in vitro* stimulations might lead to the differences of T cell-mediated immune responses.

Boosting BCG-primed immune responses with subunit vaccines is expected to induce long-lived memory T cells. T_SCM_ and T_CM_ provide long-term immune protection against *M. tuberculosis* infection (8, 56–58). Treating BCG-immunized mice with Suplatast tosylate and D4476, inhibitor of T help 2 and regulatory T cells, favored the development of T_CM_ over T_EM_. Adoptively transfer of T_CM_ cells generated by treatment with immunomodulators during BCG vaccination conferred protective efficiency against *M. tuberculosis* infection (59). To improve the access of BCG antigens to MHC I pathway, a urease C-deficient recombinant BCG ΔureC::hly (rBCG ΔureC::hly), which secreted pore-forming listeriolysin (Hly) was constructed (60, 61). rBCG ΔureC::hly immunization produced greater expansion of T_CM_ than BCG (62). Transfer of antigen-specific T_CM_ distinctly provided protection against *M. tuberculosis* infection (62). The Ag85B-ESAT-6/CAF01 subunit vaccines could promote long-term protective immune responses characterized by high levels of multifunctional T cells with proliferative potential (63). Mice immunized with ID93/GLA-SE exhibited a significant reduction of *M. tuberculosis* and elicited sustained antigen-specific multifunctional IFN-γ, tumor necrosis factor alpha (TNF-α), and IL-2 co-producing CD4^+^ T cells (64). A novel Sendai virus vectored TB vaccine (SeV85AB) induced antigen-specific T_CM_ cells and enhanced BCG-primed immune protection (65).

It is well-known that T_eff_ is apoptotic at 1-2 weeks after immunization or infection (66), T_EM_ cells wane around 90 days (42), T_CM_ and T_SCM_ cells live for a long time after formation (67, 68). In our previous study we found that at 25 weeks after the last immunization, the immune responses of T_EM_ were undetectable, but the immune response of long-lived memory T cells, including T_CM_ and T_SCM_, could be detected by restimulation with antigen (41). At 12 weeks after vaccination T cell subsets in spleen could include T_EM_, T_CM_ and T_SCM_ cells, but only T_CM_ and T_SCM_ cells could maintain up to 28 weeks. In this study, the vaccine-induced immune responses at 12 and 28 weeks after MH/EC boosting were detected. At 12 weeks after last immunization, EC immunizations thrice at 12-16-24 weeks group produced high numbers of IL-2 producing CD4^+^ and CD8^+^ T cells. MH boosting twice at 12-24 weeks produced more IFN-γ producing CD4^+^ T cells than MH boosting thrice at 12-16-24 weeks and EC immunizations twice at 12-24 weeks. As same as the results of cytokines production, the results of memory T cells proliferation showed that both programs of MH boosting at 12-24 weeks and EC immunizations at 12-16-24 weeks improved the proliferation of long-lived memory T cells. At 28 weeks after last immunization, proliferation assay, cultured ELISPOT assay, detection of IFN-γ production following antigen restimulation *in vivo* and *in vitro* were applied for the detection of vaccine-induced long-lived memory T cells. The results showed that both MH boosting at 12-24 weeks and EC immunizations at 12-16-24 weeks enhanced the number and function of long-lived memory T cells. In the protection efficiency against *M. tuberculosis* H37Ra, these two regiments of MH boosting at 12-24 weeks and EC immunizations at 12-16-24 weeks prolonged BCG-primed protective efficacy, consisting with the assumption that vaccine-generated memory T cells were essential for preventing or limiting *M. tuberculosis* infections (69).

The times of antigen stimulations affect the development of memory T cells (70–73). It was reported that the expansion and survival of memory T cell populations were impaired if antigens were stimulated more times (33, 74). The decreasing-potential model for generating effector and memory T cell heterogeneity suggests that repetitive stimulation with antigen and other signals drive greater effector cell proliferation and terminal differentiation (32). Our experiments found that following BCG priming, the RD antigen EC immunizations thrice at 12-16-24 weeks induced long-term immune protection. Claus Aagaard et al. reported that the *M. tuberculosis*-specific (or “non-BCG”) vaccine ESX-1-associated antigens (H74) boosting BCG-primed mice three times at 2-week intervals added significantly to the BCG-induced protection (75). BCG expresses the antigen of Mtb10.4 and HspX. In the program of MH boosting twice, Mtb10.4 and HspX actually encountered three times, which was as same times as that of ESAT6 and CFP10 encountered in the EC immunizations thrice program. BCG-prime and MH boosting twice induced more long-lived memory T cells than MH boosting thrice. In a clinical trial in which BCG-vaccinated participants received varying doses of ID93 + GLA-SE at 0-28-112 days, the vaccination-induced Th1 cellular responses peaked after two administrations rather than after the third administration (74). It suggests that the immune schedule including vaccination times and intervals be related to the production of memory T cells, and should be investigated for different vaccines respectively.

In our study, MH boosting at 8-14 weeks and EC immunizations at 8-10-14 weeks after BCG priming decreased the long-term protective efficiency. The interval of vaccination affected the generation of T_CM_ (25). Subunit vaccine boosting at short intervals might produce abundant Teff/T_EM_ cells, which wane several weeks (47) and are poised for immediate protection at the expense of forming stable long-term memory (24). Therefore, in the case of vaccine boosting, a suitable long interval played an important role in inducing long-term immune memory.

As far as humoral immune responses were considered, BCG-prime and subunit vaccine MH/EC immunizations improved the production of durable antigen-specific antibodies, while BCG revaccination did not stimulate the production of durable antibodies. The same results were also observed in clinical trials on H4:IC31 and H56:IC31. In the BCG-primed population, H4:IC31 and H56:IC31 vaccine boosting significantly increased the IgG level, while BCG revaccination did not (76). In addition, BCG revaccination of cattle did not increase the level of antigen-specific antibodies (77). Mounting data showed that the subunit vaccines such as ID93 + GLA-SE (74, 78) and M72/AS01 (79, 80) vaccination increased antigen-specific IgG responses significantly in the animal experiments and clinical trials. The role of antibodies in immune protection against *M. tuberculosis* infection needs further investigation (81, 82).

In this study, H37Ra was used to preliminarily evaluate the protective efficacy induced by fusion proteins MH and EC in adjuvant DP with different boosting schedules. Our study suggests that both BCG and “non-BCG” antigens require different schedules to boost BCG-primed immune responses so as to induce long-term immune protection against TB. Although H37Ra has some limitations, it still has been used to preliminarily evaluate the protective efficacy of vaccines (83). However, H37Ra was an attenuated strain and could not persist in mice for a long time as virulent strain. For this reason, we only detected bacterial load in lung of the mice after intranasal challenge. The whole lungs of each mouse were used for bacteria counting and the pathological lesion was not analyzed. In future, virulent *M. tuberculosis* strain H37Rv will be required to evaluate the protective efficacy induced by BCG-prime and different subunit vaccines-boost with different strategies.

## 5 Conclusion

Following BCG priming, MH boosting twice at 12-24 weeks, and EC immunizations thrice at 12-16-24 weeks could produce long-term immune responses and improved the BCG-primed protective efficiency. MH represents the antigens from BCG, while EC represents the antigens from RD. It suggests that following BCG-priming BCG antigen MH boosted twice or “non-BCG” antigens EC immunized thrice at suitable intervals tend to induce long-lived memory T cells. This finding will be helpful for optimizing subunit vaccine boosting schedules to prolong BCG-primed immune protection. Following BCG vaccination, the expression and persistence of antigens *in vivo* can vary, therefore the boosting schedules of different subunit vaccines should be investigated respectively to induce durable immunity against *M. tuberculosis* infection.

## Data Availability Statement

The original contributions presented in the study are included in the article/**Supplementary Material**. Further inquiries can be directed to the corresponding author.

## Ethics Statement

The animal study was reviewed and approved by Institutional Animal Care and Use Committee of Lanzhou University.

## Author Contributions

BZ designed experiments. WL, PH, YLM, DT, FL, TX, JH, JW, YJM, and HN performed experiments. WL and BZ wrote and revised the manuscript. All authors have read and agreed to the published version of the manuscript.

## Funding

The work was funded by National Key Research and Development Program of China (2021YFC2301503), National Science and Technology Major Projects of China (2018ZX10302302-002-003), Gansu Science and Technology Project (21JR7RA534) and National Science Foundation of China (31470895).

## Conflict of Interest

The authors declare that the research was conducted in the absence of any commercial or financial relationships that could be construed as a potential conflict of interest.

## Publisher’s Note

All claims expressed in this article are solely those of the authors and do not necessarily represent those of their affiliated organizations, or those of the publisher, the editors and the reviewers. Any product that may be evaluated in this article, or claim that may be made by its manufacturer, is not guaranteed or endorsed by the publisher.
