# Supplementary material for: Optimizing the Boosting Schedule of Subunit Vaccines Consisting of BCG and “Non-BCG” Antigens to Induce Long-Term Immune Memory
Source: Front Immunol. 2022 Apr 12;13:862726. doi: 10.3389/fimmu.2022.862726 (PMC9039131; doi:10.3389/fimmu.2022.862726)
Supplement: Supplementary file 1 [file DataSheet_1.docx]

**Supplementary materials:**

**Supplementary Table1. Endotoxin concentration in fusion proteins**

| **Proteins** | **Concentration of**  **endotoxin (EU/****μg)** | **Standard curve** |
| --- | --- | --- |
| Mtb10.4-HspX | 0.009031 | Y = 1.9271X - 0.0662 R² = 0.9977 |
| ESAT6-CFP10 | 0.00985 |  |

The concentrations of endotoxin in fusion proteins were detected by Limulus amebocyte lysate (LAL). In standard curve, Y: Concentration(EU/ml), X: OD 545nm.


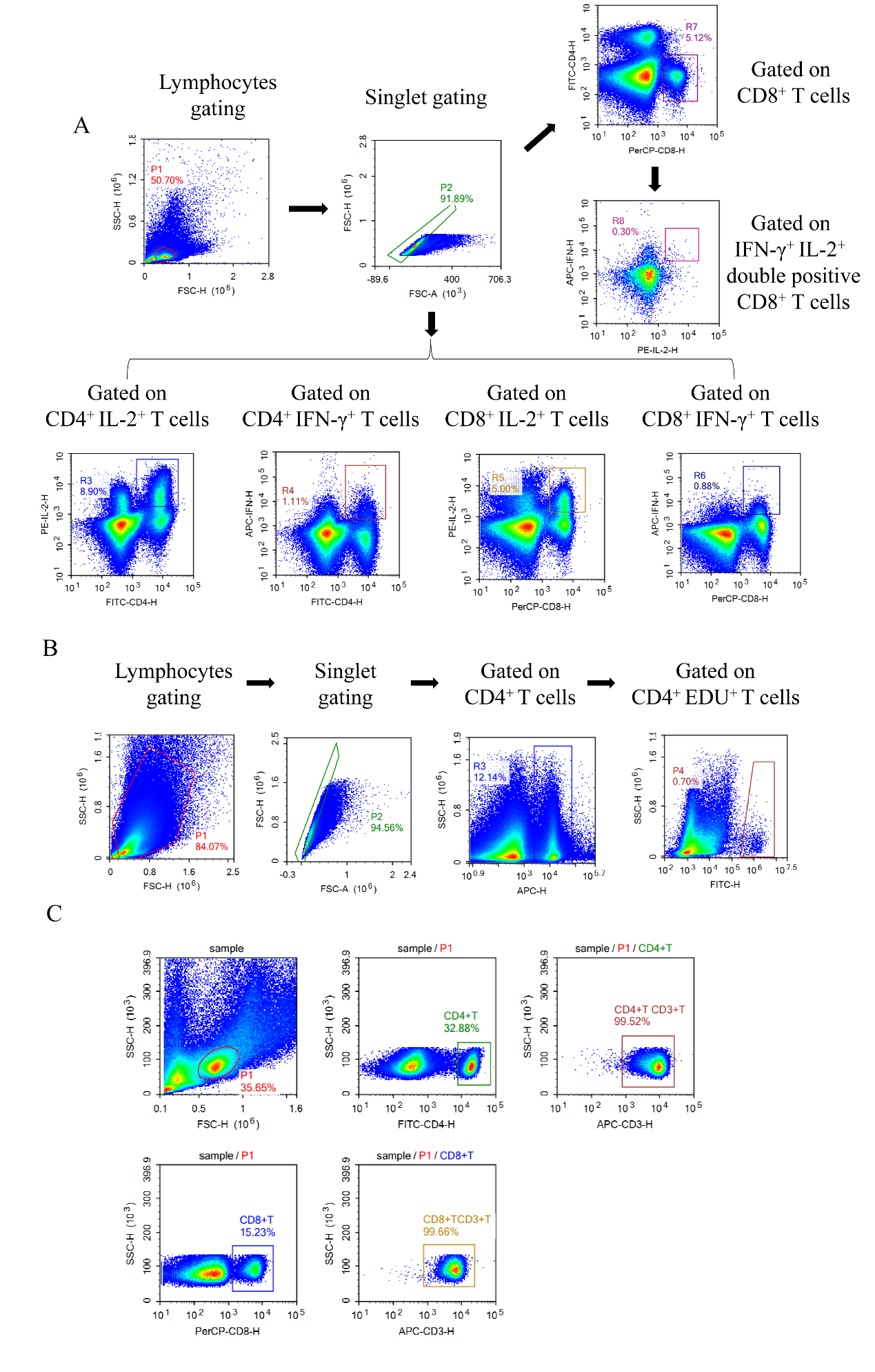


**Supplementary Figure 1 Flow cytometry gating strategy. (A)** In intracellular cytokine staining assay, spleen lymphocytes were stained with the anti-CD4-FITC, anti-CD8-PerCP-Cy5.5, anti-IFN-γ-APC and anti-IL-2-PE. Lymphocytes were first gated by the parameters SSC-H and FSC-H (lymphocytes), and then single cells were gated by the parameters FSC-H and FSC-A (single cells). Finally, CD4^+^ IFN-γ^+^ T cells, CD4^+^ IL-2^+^ T cells, CD8^+^ IFN-γ^+^ T cells, CD8^+^ IL-2^+^ T cells and CD8^+^ IFN-γ^+^ IL-2^+^ T cells were analyzed by flow cytometric. **(B)** In EdU assays, spleen lymphocytes were stained with the APC-CD4. Lymphocytes were first gated by the parameters SSC-H and FSC-H (lymphocytes), and then single cells were gated by the parameters FSC-H and FSC-A (single cells). Finally, the CD4^+^ EdU^+^ T cells were analyzed by flow cytometric. **(C)** The spleen lymphocytes separated by Mouse 1 × Lymphocyte Separation Medium (Dakewe Biotech Company Limited, China) were stained with the anti-CD3-APC (145-2C11, Biolegend), anti-CD4-FITC (RM4-5, eBioscience) and anti-CD8-PerCP-Cy5.5 (53-6.7, eBioscience). The CD4^+^ and CD8^+^ single-positive cells and CD3^+^ CD4^+^ and CD3^+^ CD8^+^ double-positive cells were analysis by flow cytometry.


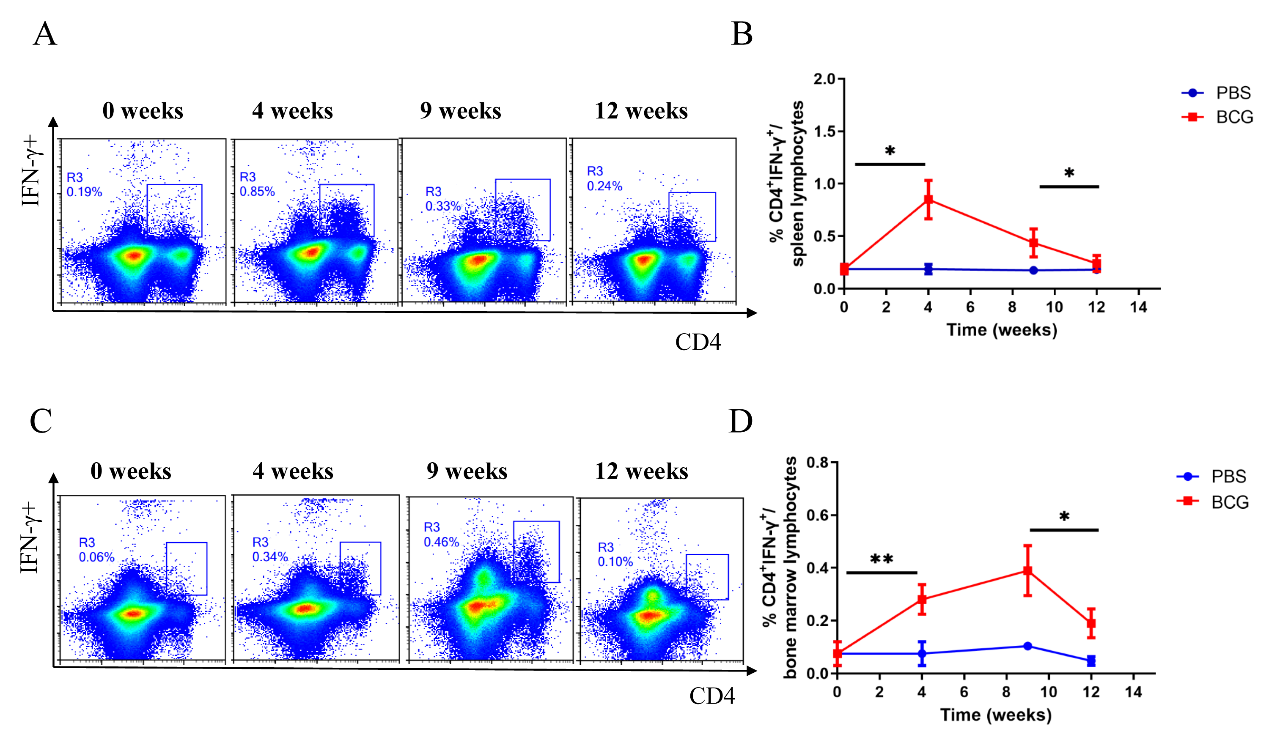


**Supplementary Figure 2 Longitudinal changes of T cell immune response after BCG vaccination.** The spleen or bone marrow lymphocytes were separated at 4, 9, 12 weeks after BCG immunization. And lymphocytes were stimulated with PPD (4ug/ml) *in vitro* for 12 hours. The intracellular cytokines staining was analyzed using flow cytometry. **(A)** Representative flow cytometric analysis of IFN-γ producing CD4^+^ T cells in spleen lymphocytes. **(B)** Statistical analysis of IFN-γ producing CD4^+^ T cells in spleen lymphocytes. **(C)** Representative flow cytometric analysis of IFN-γ producing CD4^+^ T cells in bone marrow lymphocytes. **(D)** Statistical analysis of IFN-γ producing CD4^+^ T cells in bone marrow lymphocytes. Results are presented as means ± SD, *n* = 4 ~ 5. The data were evaluated with unpaired two-tailed Student′s *t*-tests to compare two groups and one-way analysis of variance (ANOVA) followed by a Tukey post hoc test to compare multiple groups.* *P* < 0.05, ** *P* < 0.01.


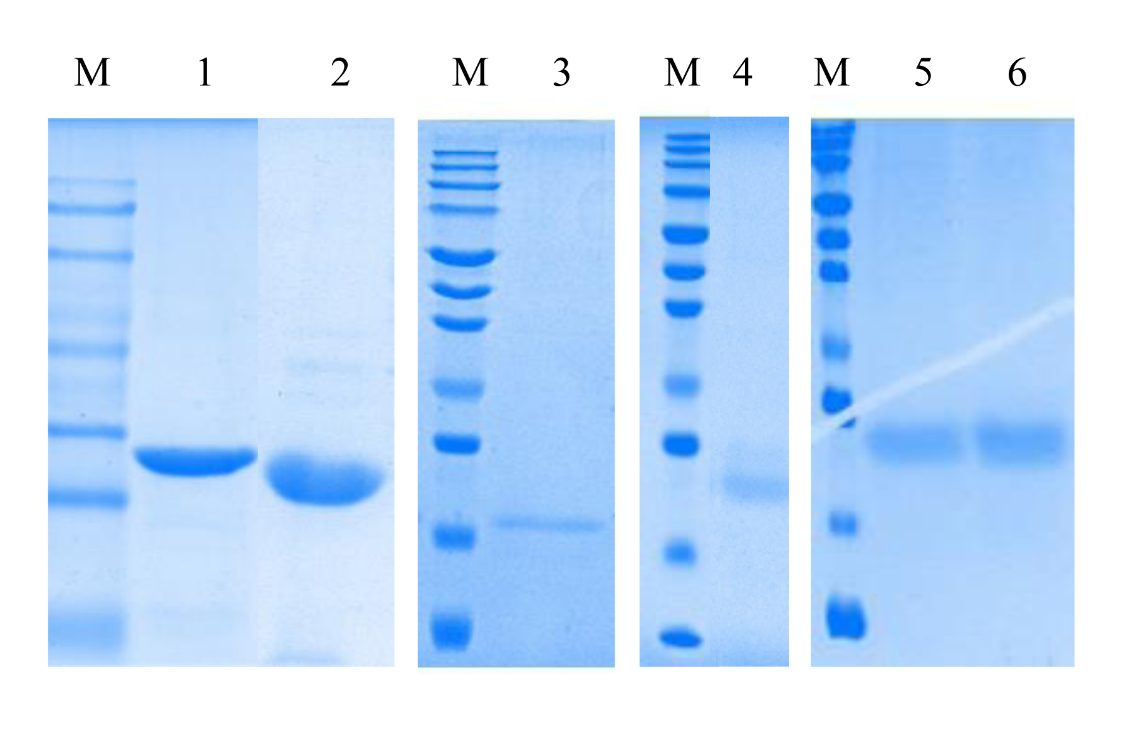


**Supplementary Figure 3** **Purification results of Mtb10.4-HspX, ESAT6-CFP10, ESAT6, CFP10 and HspX.** MH fusion antigen without tag was purified by Butyl HP and Q HP column. The fusion antigen EC without tag was purified by Q HP column. Single proteins HspX, CFP10 and ESAT6 with His tag were purified by Ni-NTA His column. M, protein molecular weight standard; 1, Purification result of Mtb10.4-HspX; 2, Purification result of ESAT6-CFP10; 3, Purification result of ESAT6; 4, Purification result of CFP10; 5-6, Purification result of HspX.


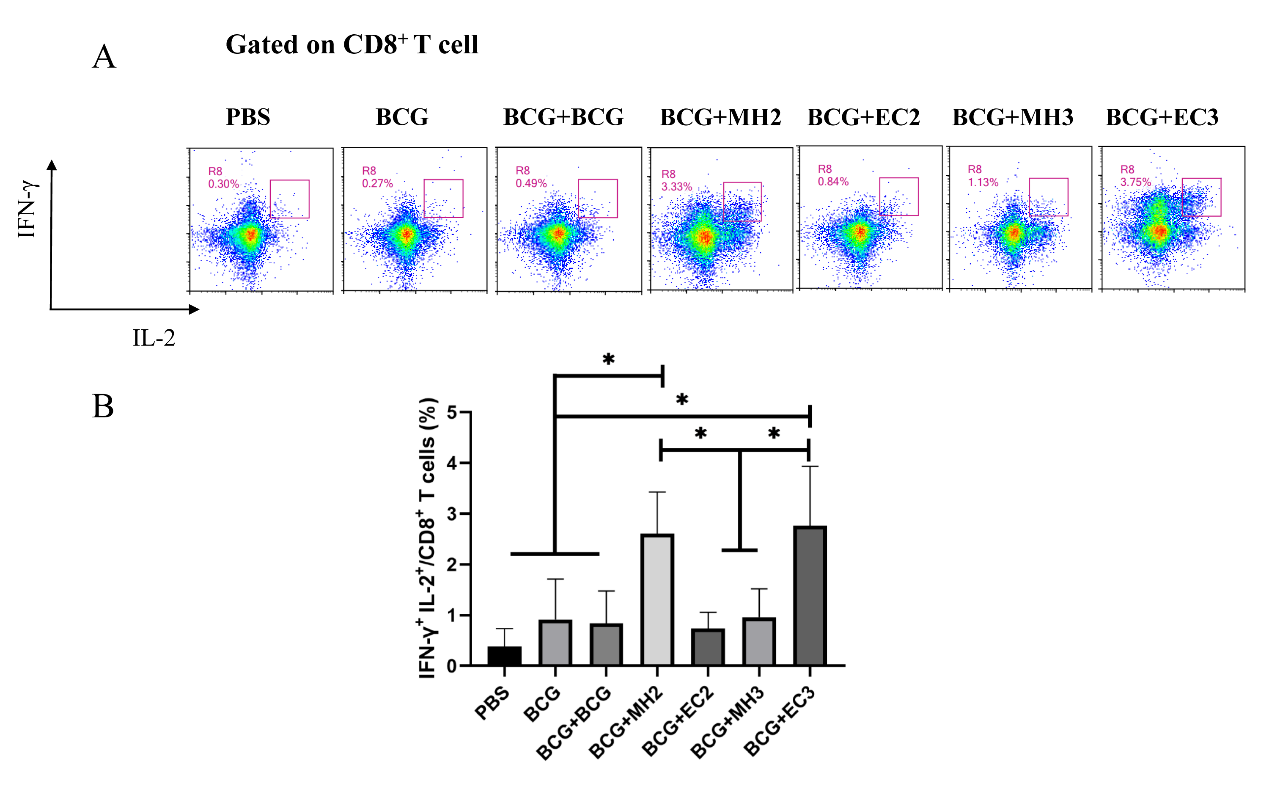


**Supplementary Figure 4** **The ratio of cytokine-producing double-positive cells following antigen stimulation.** At 12 weeks after the last immunization, the splenic lymphocytes were separated and stimulated with mixed antigens of PPD, ESAT-6, CFP10 and HspX (PHEC) *in vitro* for 12 hours. The intracellular cytokines staining was analyzed using flow cytometry. **(A)** Flow cytometric analysis of IFN-γ and IL-2 double-positive cytokine-producing CD8^+^ T cells. **(B)** Statistical analysis of the proportion of IFN-γ and IL-2 double-positive cytokine-producing CD8^+^ T cells. Results are presented as means ± SD, *n* = 4 ~ 5. The data were evaluated with unpaired two-tailed Student′s *t*-tests to compare two groups and one-way analysis of variance (ANOVA) followed by a Tukey post hoc test to compare multiple groups. * *P* < 0.05.


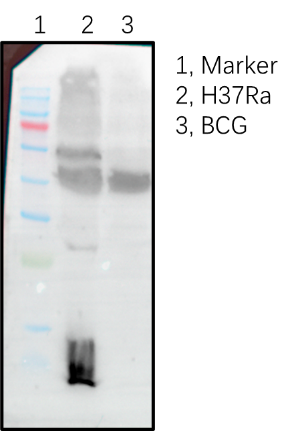


**Supplementary Figure 5** **The ESAT6 expression in H37Ra strain.** The expression of ESAT6 in H37Ra strain was detected by western blotting. The primary antibody was the sera of mice immunized with ESAT6 antigen, and the dilution ratio was 1:3000.


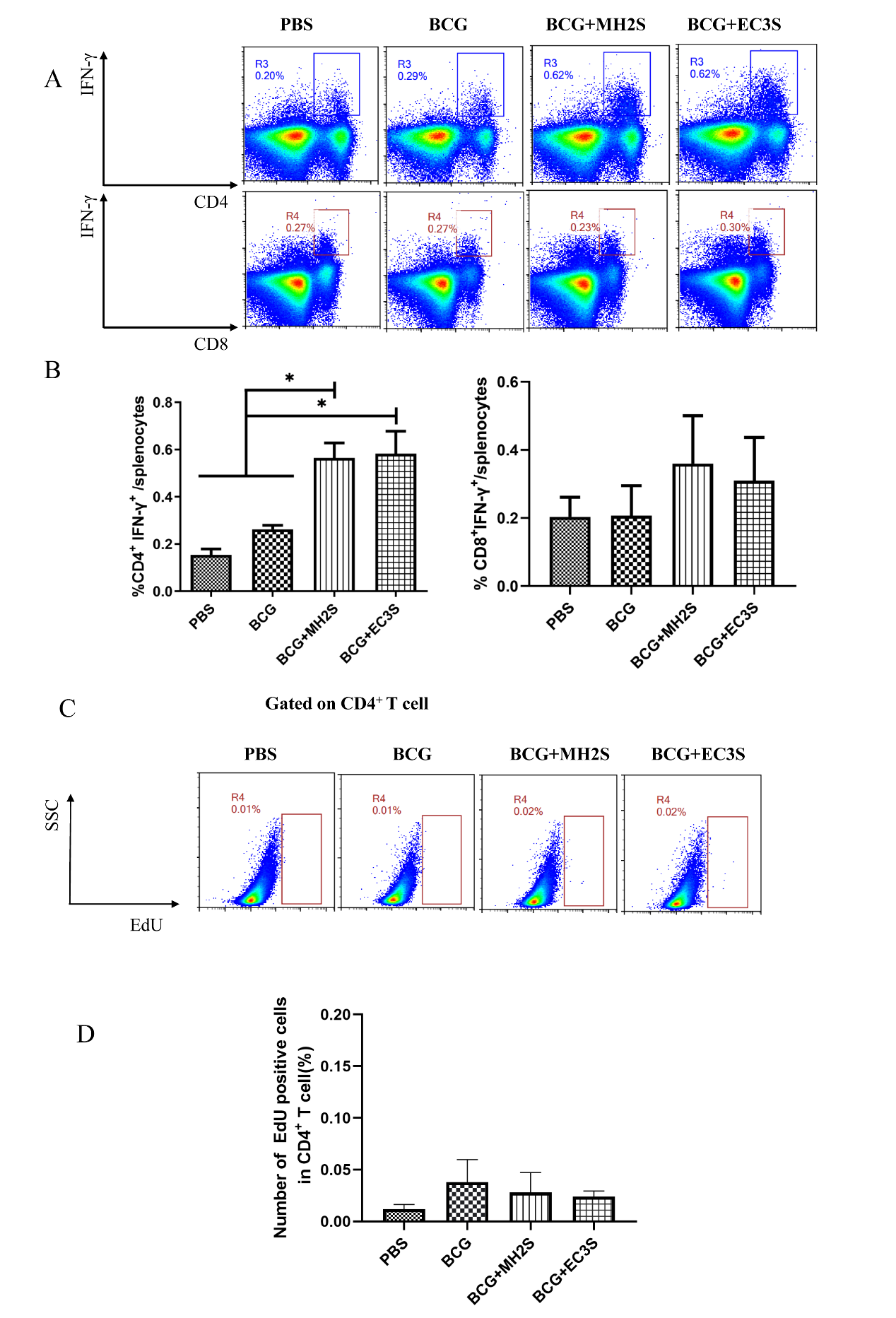


**Supplementary Figure 6** **The ratio of cytokine-producing cells and T cells proliferation in the shortened schedules.** In the shortened protocol, at 12 weeks after the last immunization, splenic lymphocytes were separated and stimulated with the mixed antigens PPD, HspX, ESAT6 and CFP10 *in vitro* for 12 hours, and the proportions of IFN-γ-producing CD4^+^ T and CD8^+^ T cells and T cell proliferative capacity were analyzed by flow cytometry. **(A)** Flow cytometric analysis of IFN-γ producing CD4^+^ T and CD8^+^ T cells. **(B)** Statistical analysis of the proportion of IFN-γ producing CD4^+^ T and CD8^+^ T cells. **(C)** Flow cytometric analysis of CD4^+^ T cells proliferation. **(D)** Statistical analysis of CD4^+^ T cell proliferation. Results are presented as means ± SD, *n* = 4 ~ 5. The data were evaluated with unpaired two-tailed Student’s *t*-tests to compare two groups and one-way analysis of variance (ANOVA) followed by a Tukey post hoc test to compare multiple groups. * *P* < 0.05.
